# The Impact of the COVID-19 Pandemic on Internet Use and the Use of Digital Health Tools: Secondary Analysis of the 2020 Health Information National Trends Survey

**DOI:** 10.2196/35828

**Published:** 2022-09-19

**Authors:** Billy Zeng, Natalie A Rivadeneira, Anita Wen, Urmimala Sarkar, Elaine C Khoong

**Affiliations:** 1 School of Medicine University of California San Francisco San Francisco, CA United States; 2 Division of General Internal Medicine at Zuckerberg San Francisco General Hospital Department of Medicine University of California San Francisco San Francisco, CA United States; 3 Center for Vulnerable Populations at Zuckerberg San Francisco General Hospital Department of Medicine University of California San Francisco San Francisco, CA United States; 4 Department of Nutrition University of California Davis Davis, CA United States

**Keywords:** COVID-19, digital divide, eHealth, telehealth

## Abstract

**Background:**

The COVID-19 pandemic increased the use of digital tools in health care (eg, patient portal, telemedicine, and web-based scheduling). Studies have shown that older individuals, racial/ethnic minority groups, or populations with lower educational attainment or income have lower rates of using digital health tools. Digitalization of health care may exacerbate already existing access barriers in these populations.

**Objective:**

This study evaluated how use of digital tools to asynchronously communicate with clinicians, schedule appointments, and view medical records changed near the beginning of the pandemic.

**Methods:**

Using 2020 Health Information National Trends Survey (HINTS) data, we examined internet use and 7 digital health technology use outcomes (electronic communication with a provider, electronic appointment scheduling, electronic test result viewing, patient portal access, portal use to download health records, portal use for patient-provider communication, and portal use to view test results). The HINTS surveyors designated surveys received after March 11, 2020, as postpandemic responses. Using weighted logistic regression, we investigated the impact of the pandemic after adjusting for sociodemographic traits (age, race/ethnicity, income, education, and gender), digital access (having ever used the internet and smartphone/tablet ownership), and health-related factors (insurance coverage, caregiver status, having a regular provider, and chronic diseases). To explore differences in changes in outcomes among key sociodemographic groups, we tested for significant interaction terms between the pandemic variable and race/ethnicity, age, income, and educational attainment.

**Results:**

There were 3865 respondents (1437 prepandemic and 2428 postpandemic). Of the 8 outcomes investigated, the pandemic was only significantly associated with higher odds (adjusted odds ratio 1.99, 95% CI 1.18-3.35) of using electronic communication with a provider. There were significant interactions between the pandemic variable and 2 key sociodemographic traits. Relative to the lowest income group (<US $20,000), the highest income group (≥US $75,000) had increased growth in the odds of ever having used the internet in postpandemic responses. Compared to the most educated group (postbaccalaureates), groups with lower educational attainment (high school graduates and bachelor’s degree) had lower growth in the odds of using electronic communication with a provider in postpandemic responses. However, individuals with less than a high school degree had similar growth to the postbaccalaureate group in using electronic communication with a provider.

**Conclusions:**

Our study did not show a widespread increase in use of digital health tools or increase in disparities in using these tools among less advantaged populations in the early months of the COVID-19 pandemic. Although some advantaged populations reported a greater increase in using the internet or electronic communication with a provider, there were signs that some less advantaged populations also adapted to an increasingly digital health care ecosystem. Future studies are needed to see if these differences remain beyond the initial months of the pandemic.

## Introduction

In response to the COVID-19 public health emergency, many American health centers transitioned to telemedicine almost overnight, with most visits conducted over the phone or video and only a limited number of visits were conducted in person [1]. As more health care became web-based, digital health tools became even more important [2]. However, studies have shown that individuals who are older, identify as persons of color, or have lower educational attainment or income are less likely to use a variety of digital health tools (eg, mobile health apps, telemedicine, and web-based medical records) [3-5].

Many of these differences in the use of digital health tools stem from structural factors—including the cost of internet access, broadband infrastructure, and digital literacy skills [6,7]—that were largely unaddressed during the pandemic. Advocates for health care equity therefore worried that requiring health care services to be accessed through digital tools without interventions to address structural barriers to digital equity in diverse populations would exacerbate preexisting differences in the use of digital health tools [8].

Although much of the focus on digital health equity since the start of the COVID-19 pandemic has been on web-based visits (or telemedicine) and increasingly remote patient monitoring tools, digital tools support a variety of other health-related tasks. Digital health technologies have been defined to include “mobile health (mHealth), health information technology, wearable devices, telehealth and telemedicine and personalized medicine” [8]. Patients can perform many health care–related tasks using digital health technologies; for example, patients can use digital tools to asynchronously communicate with clinicians, view their web-based medical records or test results, or schedule appointments.

The Health Information National Trends Survey (HINTS) is a nationally administered annual survey from the National Cancer Institute, which collects information about health communication, including patients’ use of technology for health care–related tasks outside of web-based visits [9,10]. Beginning in 2008, HINTS began including questions related to the use of the internet and other digital tools for supporting health care–related tasks. Therefore, the HINTS survey data provided an opportunity to gain insights into whether digital equity concerns were created in the early stages of the pandemic for non–telemedicine-related digital health tasks (eg, messaging with a clinician, scheduling appointments, and viewing web-based medical records).

Using HINTS data, we investigated whether disparities increased in the use of digital tools to conduct health care–related tasks after the start of the COVID-19 public health emergency. (Within this paper, we will use the term “disparity” to describe differences between groups.)

We focused on 4 sociodemographic factors previously documented to be associated with disparities in using digital health tools: age, race/ethnicity, education, and income [3-5,11-13]. We specifically hypothesized that in the early stages of the pandemic during which HINTS 2020 data were collected, there may have been increased disparities in the use of the internet and digital tools for health care–related tasks, with lower use in populations who are older, are in racial/ethnic minority groups, have lower educational attainment, or have lower income.

## Methods

### Survey Administration

Details about the HINTS administration and design are publicly available [10]. In brief, English and Spanish surveys are sent out randomly to US residential addresses and returned via mail or the internet. HINTS collects information on internet use and the use of digital tools to conduct health care tasks such as communicating with doctors, making appointments, and viewing test results [10]. We used HINTS 5 cycle 4 data, which were collected between February and June 2020 [14]. These surveys were sent to a random sample of addresses with an oversampling of areas with high minority populations to increase precision for inference on minority populations. Survey sample weights are provided in the data to allow for inferences about the whole US population. The 2020 HINTS included a variable to indicate if the survey was returned before or after the COVID-19 pandemic; surveys received after March 11, 2020, were flagged by the HINTS surveyors as postpandemic. The survey response rate for HINTS in 2020 was 37%, which was consistent with prior years.

### Outcomes

We selected 8 dichotomous (yes/no) outcome variables from questions about having ever used the internet and the use of digital tools for health-related tasks (see Multimedia Appendix 1). The internet use outcome asked if respondents had ever used the internet. There were 3 outcome variables that focused on the use of electronic means to talk to a doctor, make an appointment with a health care clinician, or view test results within the past 12 months. The last 4 outcome variables pertained to patient portal use: accessing their web-based patient portal, downloading health records, communicating with a provider, or viewing test results within the past 12 months. The last 3 outcome variables on the use of patient portals for various tasks were only asked of respondents who reported having accessed their patient portal.

### Model Design

To guide our analysis, we conceptualized the predictors that could impact each of these outcomes. In addition to having the pandemic as a key predictor variable in all models, we identified 3 groups of predictors (sociodemographic traits, digital access, and health-related factors) drawn from prior literature and described below [15-18]. Of note, the having ever used the internet outcome was included as 1 of the digital access predictor variables for modeling the other 7 outcomes.

### Predictor Variables and Covariates

#### The COVID-19 Pandemic

The pandemic was a key predictor variable that indicated if the survey response occurred after (survey received after March 11, 2020) or before the COVID-19 pandemic. This designation was made by the HINTS surveyors.

#### Sociodemographic Traits

The sociodemographic traits included in the model were age (18-34, 35-49, 50-64, 65-74, and ≥75 years), race/ethnicity (Asian, Black, Hispanic of any race, non-Hispanic White, and other), education (less than a high school degree, high school graduate, some college, bachelor’s degree, and postbaccalaureate), income (<US $20,000, US $20,000-$34,999, US $35,000-$49,999, US $50,000-$74,999, and >US $75,000), and gender (male and female). All predictors were categorical variables. Missing values in the income data were imputed and supplied by the HINTS data set. For the logistic regression models, the reference groups for age, race/ethnicity, education, income, and gender were the following, respectively: aged 18-34 years, non-Hispanic White, postbaccalaureate education, income<US $20,000, and male.

#### Digital Factors

There were 2 dichotomous variables included in this group: owns a tablet or smartphone and having ever used the internet. Having ever used the internet was an outcome in 1 model but was included as a covariate in the other models.

#### Health Care Factors

There were 3 dichotomous health care–related variables: functions as a caregiver for another individual, has access to a regular provider, and has insurance. We also included 1 categorical variable: the number of chronic diseases (0, 1, 2, or ≥3) based on self-reported diagnoses of depression, hypertension, diabetes, heart disease, or lung disease, with 0 chronic diseases used as the reference value.

### Analysis

We report descriptive statistics of predictor variables, covariates, and outcomes unweighted. To infer population-level statistics, we report weighted proportions using weights provided by the HINTS data set. Using weight adjusted survey data, we constructed bivariate and multivariable logistic regression models for each of the 8 outcomes. The models for all outcomes used all the predictor and covariate variables listed above; we did not conduct variable selection, since all variables have been shown to impact these outcomes in the literature.

To determine the impact of the pandemic, we focused on the pandemic variable and the interaction terms between the pandemic variable and the 4 sociodemographic traits of interest (race/ethnicity, age, education, and income). The Wald test was used to evaluate the interaction between pandemic status and these 4 sociodemographic traits. Interactions at *P*<.10 were included in the final overall model. The final overall model was used to generate marginal expected odds and SE of each outcome for each sociodemographic and pandemic interaction pair at *P*<.10.

All analyses were performed using R statistical software (version 4.1.0; R Foundation for Statistical Computing). To adjust for complex survey design, we used a survey adjustment via the *survey* package (version 4.0) to apply sampling and jackknife replicate weights [19]. The *survey* package was also used to conduct the statistical Wald test for interaction variables. All regression models were created with complete cases. The *emmeans* package (version 1.6.0) was used to compute the expected means and SEs of odds [20], and the *ggplot2* package (version 3.3.3) was used for plot generation [21].

We used *P*<.05 to determine statistical significance for all outcomes. We did not adjust for multiple hypothesis testing due to having planned few comparisons rather than every possible comparison and to avoid increasing type II error [22,23].

## Results

### Survey Respondents and Outcomes

Of the 3865 survey respondents, 1437 responded before the pandemic indicator and 2428 responded post the pandemic. Table 1 describes the survey participants and outcomes. A large portion (3148/3865, 86%) of participants reported having ever used the internet, but less than half of them reported using any of the digital health tools. The most common uses of digital tools for health care–related tasks were using electronic means to schedule a health care appointment (1891/3865, 49%) and communicate with a provider (1800/3865, 47%). Only 39% (1553/3865) of respondents reported having ever accessed their patient portal. (As noted in table 1, percentages are weighted so may not align with the n/N presented.) Of those who accessed their patient portal, 87% (1349/1553) reported viewing their test results, and 59% (920/1553) reported messaging their clinicians.

**Table 1:** Traits of included participants (N=3865).

Trait, variable^a^			2020, prepandemic (n=1437), n (weighted %^b^)	2020, postpandemic (n=2428), n (weighted %^b^)	2020, total, n (weighted %^b^)
**Sociodemographic**					
	**Gender**				
		Female	804 (47.71)	1400 (51.59)	2204 (50.22)
	**Age (years)**				
		18-34	151 (19.21)	333 (28.89)	484 (25.47)
		35-49	212 (21.93)	491 (26.37)	703 (24.80)
		50-64	433 (31.88)	709 (24.25)	1142 (26.95)
		65-74	361 (13.81)	508 (10.42)	869 (11.62)
		≥75	237 (9.74)	303 (7.61)	540 (8.36)
	**Race/ethnicity**				
		Asian	51 (3.84)	110 (5.37)	161 (4.83)
		Black	135 (7.70)	346 (11.75)	481 (10.32)
		Hispanic	170 (11.86)	426 (17.84)	596 (15.73)
		White	904 (7.37)	1229 (7.31)	2133 (7.34)
		Other	49 (4.25)	70 (2.45)	119 (3.09)
	**Income (US $)**				
		<20,000	258 (15.13)	506 (17.38)	764 (16.58)
		20,000-34,999	189 (11.02)	302 (11.73)	491 (11.48)
		35,000-49,999	180 (11.74)	336 (12.74)	516 (12.39)
		50,000-74,999	257 (17.44)	392 (17.98)	649 (17.79)
		≥75,000	547 (43.66)	880 (39.67)	1427 (41.08)
	**Education**				
		Less than a high school degree	90 (7.01)	183 (8.25)	273 (7.81)
		High school graduate	251 (19.69)	454 (23.09)	705 (21.89)
		Some college	415 (38.61)	666 (37.82)	1081 (38.10)
		Bachelor’s degree	358 (19.97)	621 (17.32)	979 (18.26)
		Postbaccalaureate	285 (12.05)	399 (10.70)	684 (11.18)
**Digital Factors**					
	Having ever used the internet		1187 (87.09)	1961 (85.09)	3148 (85.80)
	Owns a tablet or smartphone		1210 (87.63)	2029 (88.58)	3239 (88.25)
**Health Factors**					
	Has insurance		1352 (90.43)	2252 (89.42)	3604 (89.78)
	Has a regular provider		1046 (69.60)	1582 (56.91)	2628 (61.39)
	Is a caregiver		198 (14.53)	378 (16.66)	576 (15.91)
	**Number of Chronic Disease**				
		0	506 (42.58)	922 (45.45)	1428 (44.44)
		1	356 (20.76)	550 (18.98)	906 (19.61)
		2	335 (20.58)	560 (21.88)	895 (21.42)
		≥3	229 (15.71)	362 (12.79)	591 (13.82)
**Outcomes**					
	Electronic communication with a provider		659 (48.22)	1141 (45.61)	1800 (46.53)
	Electronic means to make Appointments		680 (48.78)	1211 (48.73)	1891 (48.75)
	Electronic means to view test results		634 (45.67)	995 (39.42)	1629 (41.63)
	Having ever accessed their patient portal		605 (41.18)	948 (38.57)	1553 (39.49)
	Patient portal to message a provider^c^		350 (57.85)	570 (60.13)	920 (59.24)
	Patient portal to view test results^c^		530 (87.60)	819 (86.39)	1349 (86.53)
	Patient portal to download health records^c^		171 (28.26)	284 (29.95)	455 (29.30)

^a^Each variable had less than 10% missing data.

^b^The percentage rates were calculated using weighted data to represent the US population.

^c^Patient portal tasks were only asked of those who had accessed the patient portal. Therefore, the proportions are reported only out of those that reported having ever accessed their patient portal.

### Impact of the Pandemic

All bivariate models and multivariable analysis are shown in Multimedia Appendices 2 and 3, respectively. In the adjusted analysis, older age, lower income, lower educational attainment, and race/ethnic minority groups were associated with lower odds of having ever used the internet (Multimedia Appendix 3). The same patterns for age, income, and educational attainment were seen for the other outcomes, but the findings were mixed by race/ethnicity (Multimedia Appendix 3).

After accounting for other variables, the pandemic variable was only significant for using electronic means to communicate with a provider. Postpandemic respondents had higher odds (adjusted odds ratio [aOR] 1.99, 95% CI 1.18-3.35; *P*=.01; see Multimedia Appendix 3) of using electronic means to communicate with a provider than responses from the prepandemic period.

**Figure 1:**
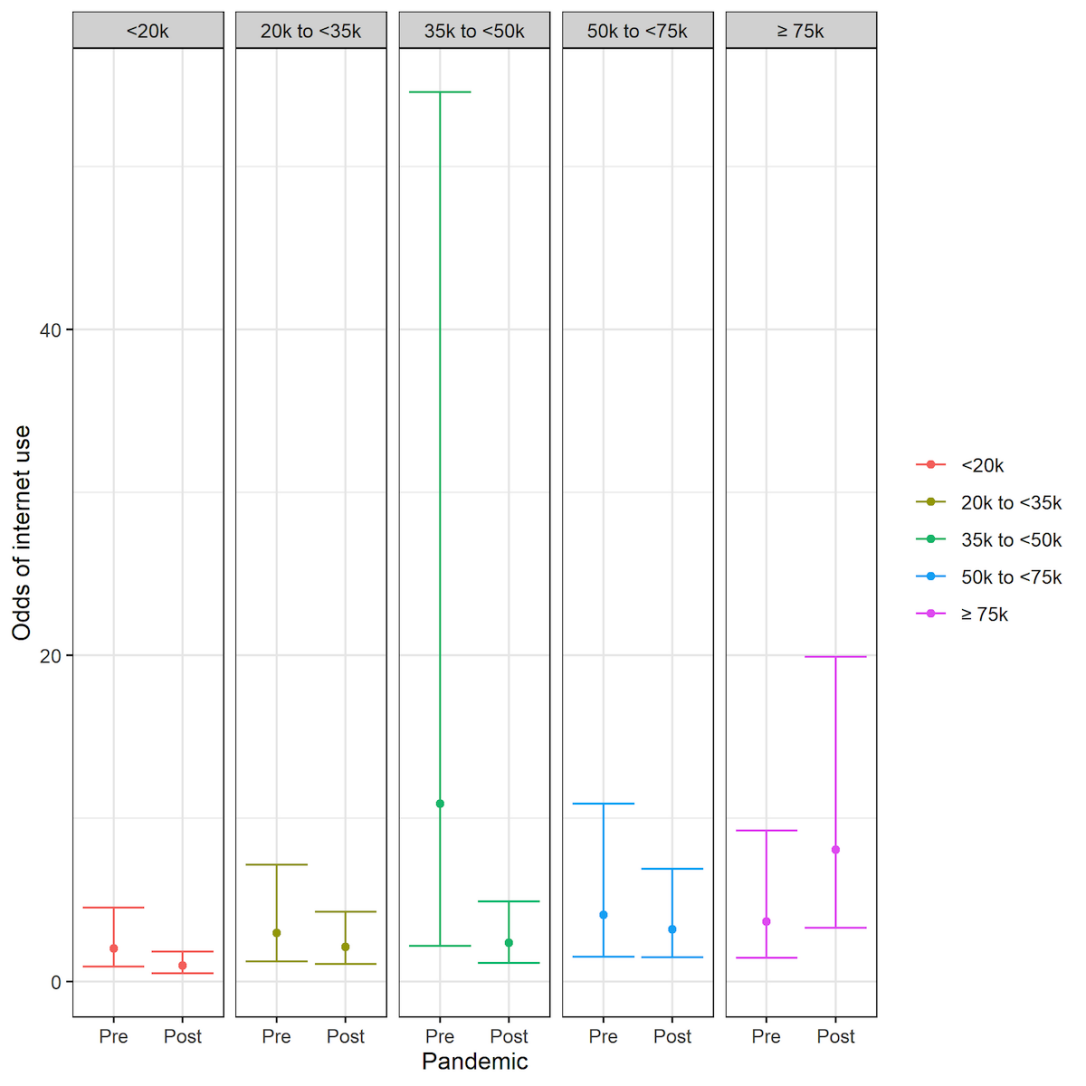
Odds of having ever used the internet before (pre) and after (post) the pandemic among income groups (in US $).

**Figure 2:**
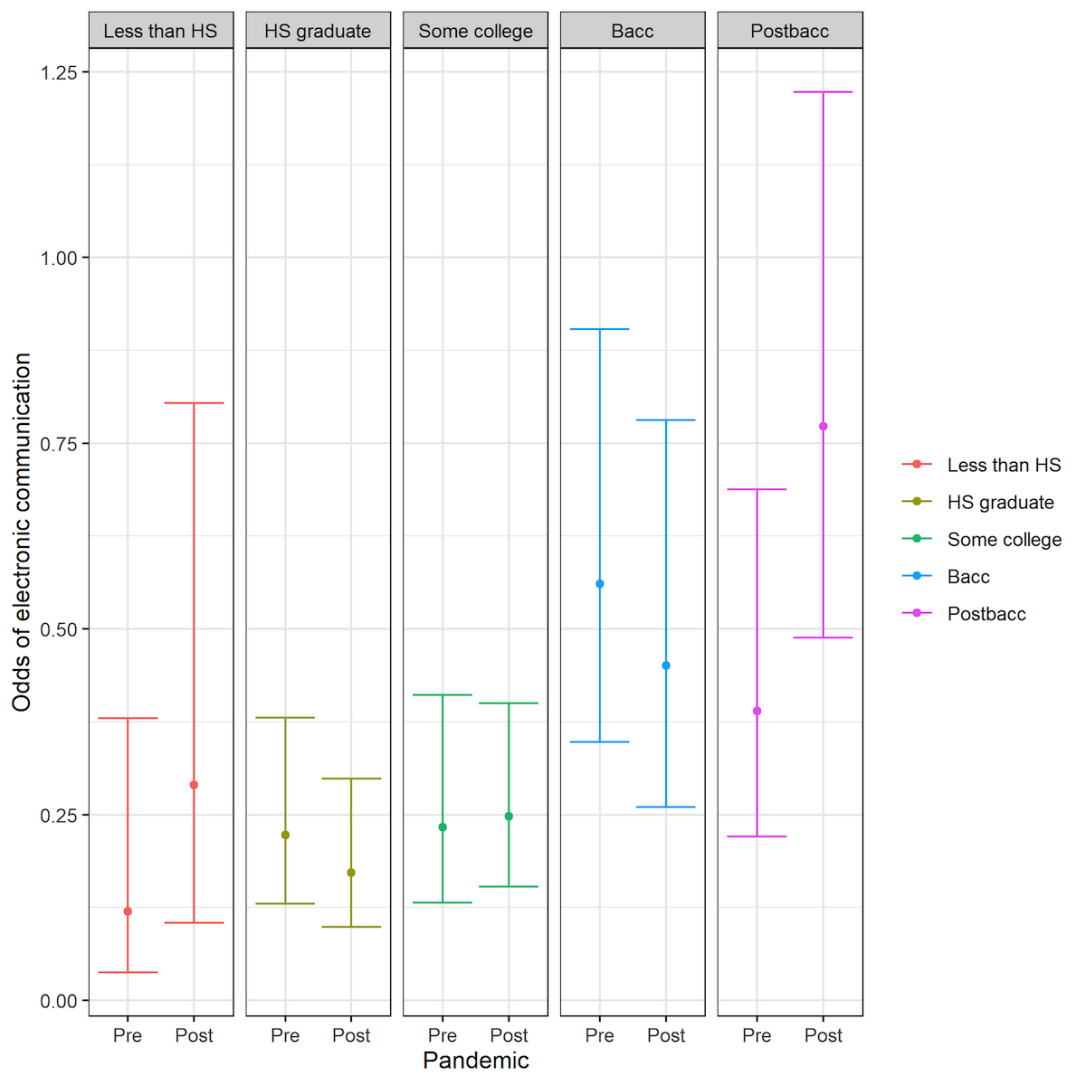
Odds of using electronic communication with a provider among different education groups before (pre) and after (post) the pandemic. Bacc: baccalaureate; HS: high school; Postbacc: postbaccalaureate.

The interaction between the pandemic variable and 4 sociodemographic variables (age, race/ethnicity, education, and income) was only significant for 2 outcomes: having ever used the internet and electronic communication with a provider (see Multimedia Appendix 3.). These significant interactions are shown in Figures 1 and 2.

For the outcome related to internet use, there was a significant interaction between the pandemic variable and income (see Figure 1). Specifically, respondents in the ≥US $75,000 income group had an increase in the odds of having ever used the internet post the pandemic, which was significantly different from the <US $20,000 income group (*P*=.02).

The use of electronic communication with a provider was notably increased in the highest educational attainment group (postbaccalaureate). As seen in Figure 2 (and detailed in Multimedia Appendix 3), the highest educated group (postbaccalaureate) had a significantly greater growth in odds of conducting this digital task than both the groups with a bachelor’s degree (*P*=.02) and high school education only (*P*=.01). There was also a trend toward greater growth compared to the group with some college education (aOR 0.54, 95% CI 0.27-1.06), but this result was not statistically significant (*P*=.07). The growth in using electronic communication with a provider in the most educated group was not significantly different from patterns seen in conducting this task by the respondents who reported less than a high school education (*P*=.80).

## Discussion

### Principal Findings

Overall, we found mixed results on how the pandemic affected internet use, the use of digital tools to communicate with clinicians or schedule appointments, and patient portal use. For most of the outcomes, there were no significant differences before and after the pandemic in the early months of the pandemic and no significant changes in disparities in the uptake of digital health tools.

Consistent with prior literature, we did find that populations with a history of digital exclusion (older, lower income, lower educational attainment, and racial/ethnic minority groups) continue to have lower odds of using the internet and a variety of digital health tools. These disparities, particularly in telemedicine use, have been repeatedly documented since the start of the pandemic [24-29]. However, unlike most other studies, we did not study telemedicine use. In prior studies, we have found that the accessibility and use of 1 digital health tool does not translate to other tools [4]; that is, the lessons learned about disparities in telemedicine use may not be applicable to disparities in patient portal use or the use of web-based communication and scheduling tools. By focusing on web-based scheduling, electronic communication, and patient portal use—tools that were relatively widely available both before and after the pandemic, we were able to explore how the pandemic immediately changed the use of these tools and if there was an increase or decrease in disparities in using these digital tools. This comparison of disparities before and after the pandemic on nontelemedicine digital health technologies is different from much of the literature that has largely provided only a static look at the disparities in telemedicine uptake post the pandemic.

With this focus in mind, we did find that immediately after the pandemic, after adjusting for other factors, there were increased odds overall in the use of electronic communication with a provider. One reason for this finding may be that the policies enacted by the Centers for Medicare and Medicaid Services to incentivize the use of telehealth [30,31] created a digital health care environment that made electronic communication more accessible to all populations. Alternatively, it may also suggest that the move to telehealth made it more necessary or important for all individuals, including those who had not previously used electronic communication with their clinicians, to use these telecommunication tools to seek health care or advice from a medical professional.

Our study had mixed findings on how differences in the uptake of these digital tools were immediately impacted by the public health emergency. Immediately after the start of the pandemic, the highest income group (≥US $75,000) had a greater rate of growth in having ever used the internet than the lowest income group (<US $20,000), suggesting a widening of the disparity between income groups. This finding may reflect that higher income earners were more likely to have jobs that could be performed remotely through the internet than lower income groups [32]. A 2019 survey from Pew Research showed that 98% of respondents with an income >US $75,000 used the internet in contrast to 82% of respondents with an income <US $30,000; given the already high rate of internet use in high-income households, it seemed that there should be little room for additional growth among high-income earners. This finding reinforces the need to ensure that structural barriers to accessing the internet for low-income households are mitigated [7].

In contrast to the findings among income groups, there was some suggestion of the gaps closing between groups with different levels of educational attainment. Both the lowest educational attainment respondents (less than high school) and highest educational attainment respondents (postbaccalaureate) had similar rates of growth in the use of electronic communication tools (eg, smartphones, internet, and email) with their doctors. However, the bachelor’s degree holders and high school graduates had decreases in the odds of using electronic communication with their doctors after the pandemic, which were significantly different from the most educated group. Together, these findings suggest that although some disparities in the use of electronic communication with clinicians were closing, others were widening. It is worth highlighting that the most vulnerable group from an educational attainment perspective (less than high school education) had a larger growth in using electronic communication tools with their clinician relative to most other respondents, which defies a frequent pattern of innovations disseminating the most slowly to the most disadvantaged.

Given the rapid move of health care to telehealth settings [1], we hypothesized there would be increased inequities after the pandemic in most of our outcomes related to the use of digital tools for health care tasks. However, we had few significant findings except for respondents who reported having ever used the internet or using electronic communication with a provider. Since these data are from early in the pandemic, these 2 digital tasks likely serve as the earliest indicators of how populations were adapting to an increasingly digital health care ecosystem. We anticipate that as more data become available, we may see more changes in the use of the other digital health technologies, including those evaluated in this study as well as other tools such as telemedicine or remote patient monitoring. Although we are somewhat reassured that in the early days of the pandemic, these data do not suggest a consistent widening of inequities between more advantaged and less advantaged populations [2,6,8,33], we also believe it is necessary to reevaluate these outcomes later in the pandemic once health care teams and patients had become more accustomed to conducting more health care tasks remotely. Researchers have documented that the use of telemedicine immediately at the beginning of the COVID-19 pandemic did not reflect the more long-term patterns on telemedicine use [34]. We suspect that similar patterns for other digital health technologies may also emerge.

We believe it important to specifically highlight that we found no changes in any of the patient portal tasks, despite patient portals being the primary digital health tool that has been adopted by health systems to increase patient engagement and care accessibility. Many health care systems already had patient portals in place and tried to use their patient portals to address health care needs during the pandemic; however, studies have repeatedly showed the significant barriers to using a patient portal, including the lack of technical skills, usability, privacy concerns, and the lack of physician encouragement [35,36]. This study suggests that even in an environment where the use of a patient portal may be even more important, patient portal products did not address patients’ needs early in the pandemic; this finding is reinforced by the multiple health care systems that found that when they used patient portals to address COVID-19–related care needs (testing and vaccine scheduling), there was inequitable access to care [37]. Since the start of the pandemic, patient portal products have attempted to become more patient-centered [38], and health care teams have increased efforts to improve access for historically excluded populations [39]; future studies should evaluate if these efforts have had the intended impact of reducing disparities in patient portal use.

### Limitations

This study has several limitations. Since the 2020 HINTS responses were collected in a 5-month period between February and June 2020, the results only reflect the early impact of the pandemic. In addition, most outcome questions inquired about electronic communication over the last 12 months, hence outcomes may be less sensitive to the immediate behavior changes resulting from the pandemic. For patient portal–related outcomes, the sample size was limited to respondents who had accessed their patient portal; therefore, there may have been inadequate power to detect statistically significant changes in patient portal use. Although the survey weights are designed to extrapolate these data to the American population, owing to the limited sample size in some subgroups, there may not be enough variability to accurately evaluate the outcomes. For example, all Asian individuals in the postpandemic group reported the use of the patient portal for viewing a test result (Multimedia Appendix 3), suggesting an inadequate diversity of HINTS respondents, and these results should be considered with caution [40]. Furthermore, some studies have shown that postpandemic survey respondents are different from prepandemic survey respondents, and therefore, caution must be used when comparing responses in this survey to those in prior HINTS cycles [41]. However, we are reassured that the response rate for this HINTS cycle was similar to prior years [14]. Despite these limitations, this study adds value to the literature by evaluating early changes in the use of digital health tools during the pandemic and focusing explicitly on changes in use among historically excluded populations.

### Conclusions

Our study finds that early within the pandemic, there was not widespread increase in the use of digital health tools or in disparities in the use of digital health tools. Although these data were only from the first 3 months of the pandemic, we did find an increase in odds of using electronic communication with a provider after the pandemic and some mixed results on whether preexisting inequities between groups in the use of digital health increased. Despite health care systems’ reliance on patient portals to increase patient access and engagement, we did not see changes in the use of patient portals during the early stages of the pandemic. These early data from the pandemic support the need to explicitly study a wide range of digital health care–related tasks. Changes in the use of 1 digital task may not translate to other health care–related digital tasks.

## Appendix

Multimedia Appendix 1 Survey questions and outcome variables.

Multimedia Appendix 2 Unadjusted bivariate models.

Multimedia Appendix 3 Multivariable models.

## Abbreviations

aOR: adjusted odds ratio
HINTS: Health Information National Trends Survey

## References

[1] Peek N, Sujan M, Scott P (2020). Digital health and care in pandemic times: impact of COVID-19. BMJ Health Care Inform.

[2] Lyles CR, Wachter RM, Sarkar U (2021). Focusing on digital health equity. JAMA.

[3] Mitchell UA, Chebli PG, Ruggiero L, Muramatsu N (2019). The digital divide in health-related technology use: the significance of race/ethnicity. Gerontologist.

[4] Khoong EC, Rivadeneira NA, Hiatt RA, Sarkar U (2020). The use of technology for communicating with clinicians or seeking health information in a multilingual urban cohort: cross-sectional survey. J Med Internet Res.

[5] Yoon H, Jang Y, Vaughan PW, Garcia M (2020). Older adults' internet use for health information: digital divide by race/ethnicity and socioeconomic status. J Appl Gerontol.

[6] Sieck CJ, Sheon A, Ancker JS, Castek J, Callahan B, Siefer A (2021). Digital inclusion as a social determinant of health. NPJ Digit Med.

[7] Rodriguez JA, Clark CR, Bates DW (2020). Digital health equity as a necessity in the 21st Century Cures Act era. JAMA.

[8] Lee P, Abernethy A, Shaywitz D, Gundlapalli A, Weinstein J, Doraiswamy PM, Schulman K, Madhavan S (2022). Digital health COVID-19 impact assessment: lessons learned and compelling needs. NAM Perspect.

[9] Hesse BW, Nelson DE, Kreps GL, Croyle RT, Arora NK, Rimer BK, Viswanath K (2005). Trust and sources of health information: the impact of the Internet and its implications for health care providers: findings from the first Health Information National Trends Survey. Arch Intern Med.

[10] About HINTS. National Cancer Institute.

[11] Davies AR, Honeyman M, Gann B (2021). Addressing the digital inverse care law in the time of COVID-19: potential for digital technology to exacerbate or mitigate health inequalities. J Med Internet Res.

[12] Mackert M, Mabry-Flynn A, Champlin S, Donovan EE, Pounders K (2016). Health literacy and health information technology adoption: the potential for a new digital divide. J Med Internet Res.

[13] Gordon NP, Hornbrook MC (2016). Differences in access to and preferences for using patient portals and other eHealth technologies based on race, ethnicity, and age: a database and survey study of seniors in a large health plan. J Med Internet Res.

[14] (2020). Health Information National Trends Survey 5 (HINTS 5) Cycle 4 Methodology Report. National Cancer Institute.

[15] Irizarry T, DeVito Dabbs Annette, Curran CR (2015). Patient portals and patient engagement: a state of the science review. J Med Internet Res.

[16] Sarkar U, Karter AJ, Liu JY, Adler NE, Nguyen R, López Andrea, Schillinger D (2011). Social disparities in internet patient portal use in diabetes: evidence that the digital divide extends beyond access. J Am Med Inform Assoc.

[17] Tarver WL, Menser T, Hesse BW, Johnson TJ, Beckjord E, Ford EW, Huerta TR (2018). Growth dynamics of patient-provider internet communication: trend analysis using the Health Information National Trends Survey (2003 to 2013). J Med Internet Res.

[18] Bangerter LR, Griffin J, Harden K, Rutten LJ (2019). Health information-seeking behaviors of family caregivers: analysis of the Health Information National Trends Survey. JMIR Aging.

[19] Lumley T (2021). survey: analysis of complex survey samples. The Comprehensive R Archive Network.

[20] Lenth RV, Buerkner P, Herve M, Jung M, Love J, Miguez F, Riebl H, Singmann H (2021). emmeans: estimated marginal means, aka least-squares means. The Comprehensive R Archive Network.

[21] Wickham H, Chang W, Henry L, Pedersen TL, Takahashi K, Wilke C, Woo K, Yutani H, Dunnington D, RStudio (2020). ggplot2: create elegant data visualisations using the grammar of graphics. The Comprehensive R Archive Network.

[22] Rothman KJ (1990). No adjustments are needed for multiple comparisons. Epidemiology.

[23] Perneger TV (1998). What's wrong with Bonferroni adjustments. BMJ.

[24] Nouri S, Khoong EC, Lyles CR, Karliner L (2020). Addressing equity in telemedicine for chronic disease management during the COVID-19 pandemic. NEJM Catal Innov Care Deliv.

[25] Khoong EC, Butler BA, Mesina O, Su G, DeFries TB, Nijagal M, Lyles CR (2021). Patient interest in and barriers to telemedicine video visits in a multilingual urban safety-net system. J Am Med Inform Assoc.

[26] Rodriguez JA, Betancourt JR, Sequist TD, Ganguli I (2021). Differences in the use of telephone and video telemedicine visits during the COVID-19 pandemic. Am J Manag Care.

[27] Chunara R, Zhao Y, Chen J, Lawrence K, Testa PA, Nov O, Mann DM (2021). Telemedicine and healthcare disparities: a cohort study in a large healthcare system in New York City during COVID-19. J Am Med Inform Assoc.

[28] Ruberto RA, Schweppe EA, Ahmed R, Swindell HW, Cordero CA, Lanham NS, Jobin CM (2022). Disparities in telemedicine utilization during COVID-19 pandemic: analysis of demographic data from a large academic orthopaedic practice. JB JS Open Access.

[29] Chang JE, Lai AY, Gupta A, Nguyen AM, Berry CA, Shelley DR (2021). Rapid transition to telehealth and the digital divide: implications for primary care access and equity in a post-COVID era. Milbank Q.

[30] Patel SY, Mehrotra A, Huskamp HA, Uscher-Pines L, Ganguli I, Barnett ML (2021). Trends in outpatient care delivery and telemedicine during the COVID-19 pandemic in the US. JAMA Intern Med.

[31] (2021). Notification of enforcement discretion for telehealth remote communications during the COVID-19 nationwide public health emergency. U.S. Department of Health & Humans Services.

[32] Bonacini L, Gallo G, Scicchitano S (2021). Working from home and income inequality: risks of a 'new normal' with COVID-19. J Popul Econ.

[33] Smith B, Magnani JW (2019). New technologies, new disparities: the intersection of electronic health and digital health literacy. Int J Cardiol.

[34] (2022). Outpatient telehealth use soared early in the COVID-19 pandemic but has since receded. Peterson-KFF Health System Tracker.

[35] Anthony DL, Campos-Castillo C, Lim PS (2018). Who isn't using patient portals and why? evidence and implications from a national sample of US adults. Health Aff (Millwood).

[36] Turner K, Clary A, Hong Y, Alishahi Tabriz A, Shea CM (2020). Patient portal barriers and group differences: cross-sectional national survey study. J Med Internet Res.

[37] Stern RJ, Rafferty HF, Robert AC, Taniguchi C, Gregory B, Khoong EC, Handley MA, Day LW, Chen E (2021). Concentrating vaccines in neighborhoods with high COVID-19 burden. NEJM Catal Innov Care Deliv.

[38] Heath S (2020). Was COVID-19 healthcare's use case for the patient portal?. PatientEngagementHIT.

[39] Craig S, Madu CR, Dalembert G (2021). Never let a pandemic go to waste: applying an equity-focused quality improvement framework to close gaps in patient portal activation. Pediatrics.

[40] Shimkhada R, Scheitler AJ, Ponce NA (2021). Capturing racial/ethnic diversity in population-based surveys: data disaggregation of health data for Asian American, Native Hawaiian, and Pacific Islanders (AANHPIs). Popul Res Policy Rev.

[41] Rothbaum J (2021). How does the pandemic affect survey response: using administrative data to evaluate nonresponse in the current population survey annual social and economic supplement. United States Census Bureau.

