# Supplementary material for: The Impact of the COVID-19 Pandemic on Internet Use and the Use of Digital Health Tools: Secondary Analysis of the 2020 Health Information National Trends Survey
Source: J Med Internet Res. 2022 Sep 19;24(9):e35828. doi: 10.2196/35828 (PMC9488546; doi:10.2196/35828)
Supplement: Multimedia Appendix 1 [file jmir_v24i9e35828_app1.docx]

Outcome Variables

| Variables | HINTS Questions | Answer Choices |
| --- | --- | --- |
| Uses Internet | Do you ever go on-line to access the Internet or World Wide Web, or to send and receive e-mail? | Yes, No |
| Uses Electronic Communication with Provider | In the past 12 months have you used a computer, smart phone, or other electronic means to use e-mail or the internet to communicate with a doctor or a doctors office? | Yes, No |
| Uses Electronic Means to Make Appointments | In the past 12 months have you used a computer, smart phone, or other electronic means to make appointments with a health care provider? | Yes, No |
| Uses Electronic Means to View Test Results | In the past 12 months have you used a computer, smart phone, or other electronic means to look up medical test results? | Yes, No |
| Ever Accessed Patient Portal^a^ | How many times did you access your online medical record in the last 12 months? | None, 1-2 times, 3-5 times, 6-9 times, 10 or more times |
| Uses Patient Portal to Message Provider | In the past 12 months have you used your online medical record to securely message health care provider and staff (for example, e-mail)? | Yes, No |
| Uses Patient Portal to View Test Results | In the past 12 months have you used your online medical record to look up test results? | Yes, No |
| Uses Patient Portal to Download Health Records | In the past 12 months have you used your online medical record to download your health information to your computer or mobile device, such as a cell phone or tablet? | Yes, No |

^a.^Converted to binary outcome for 1-2 times or more “Yes” and None as “No”.
